# Supplementary figures and images for: Comprehensive analysis of differential expression profiles via transcriptome sequencing in SH-SY5Y cells infected with CV-A16
Source: PLoS One. 2020 Nov 6;15(11):e0241174. doi: 10.1371/journal.pone.0241174 (PMC7647100; doi:10.1371/journal.pone.0241174)

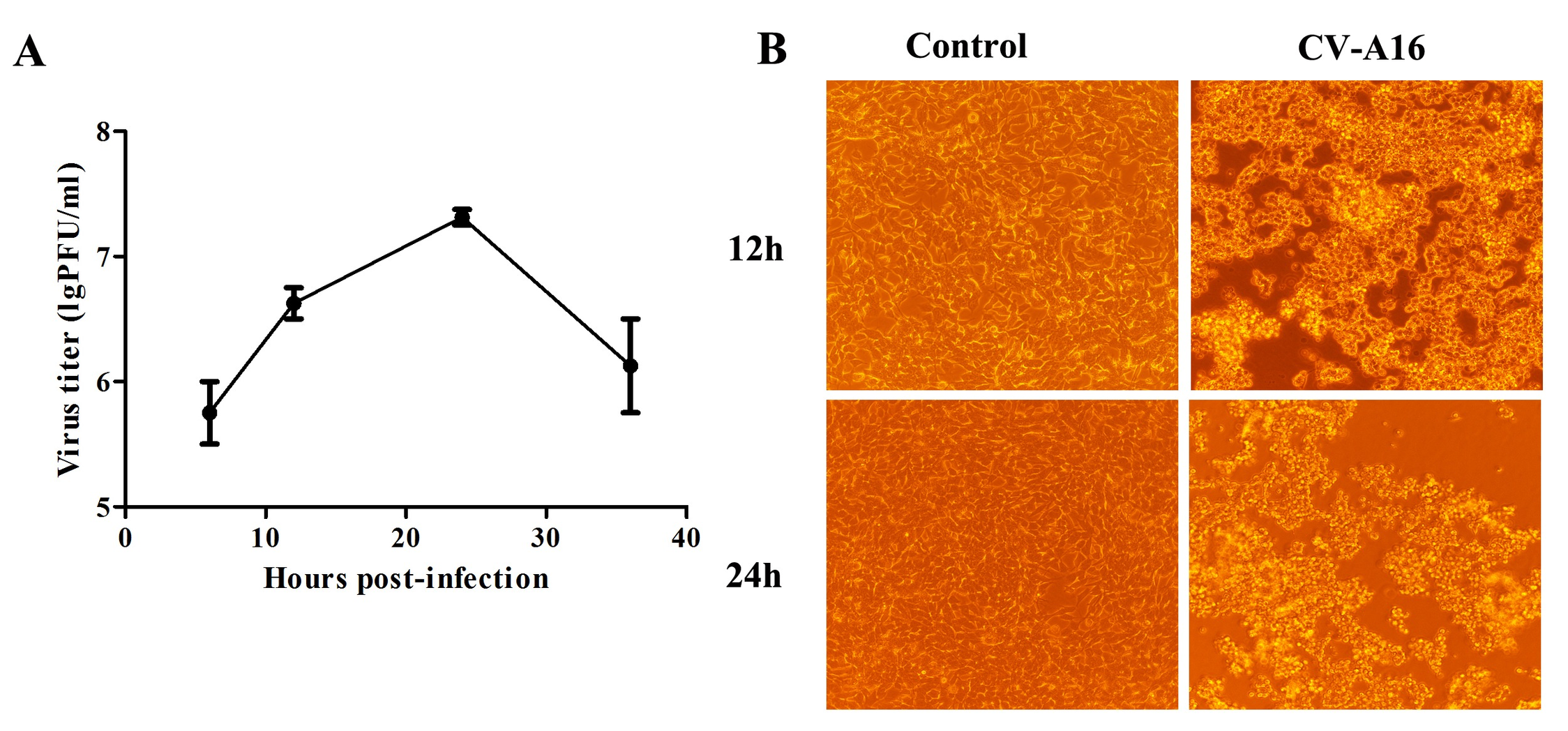

Supplement: S1 Fig — (A) The replication kinetics of CV-A16. (B) CPE of SH-SY5Y cells (200×amplication). (TIF) [file pone.0241174.s001.tif]

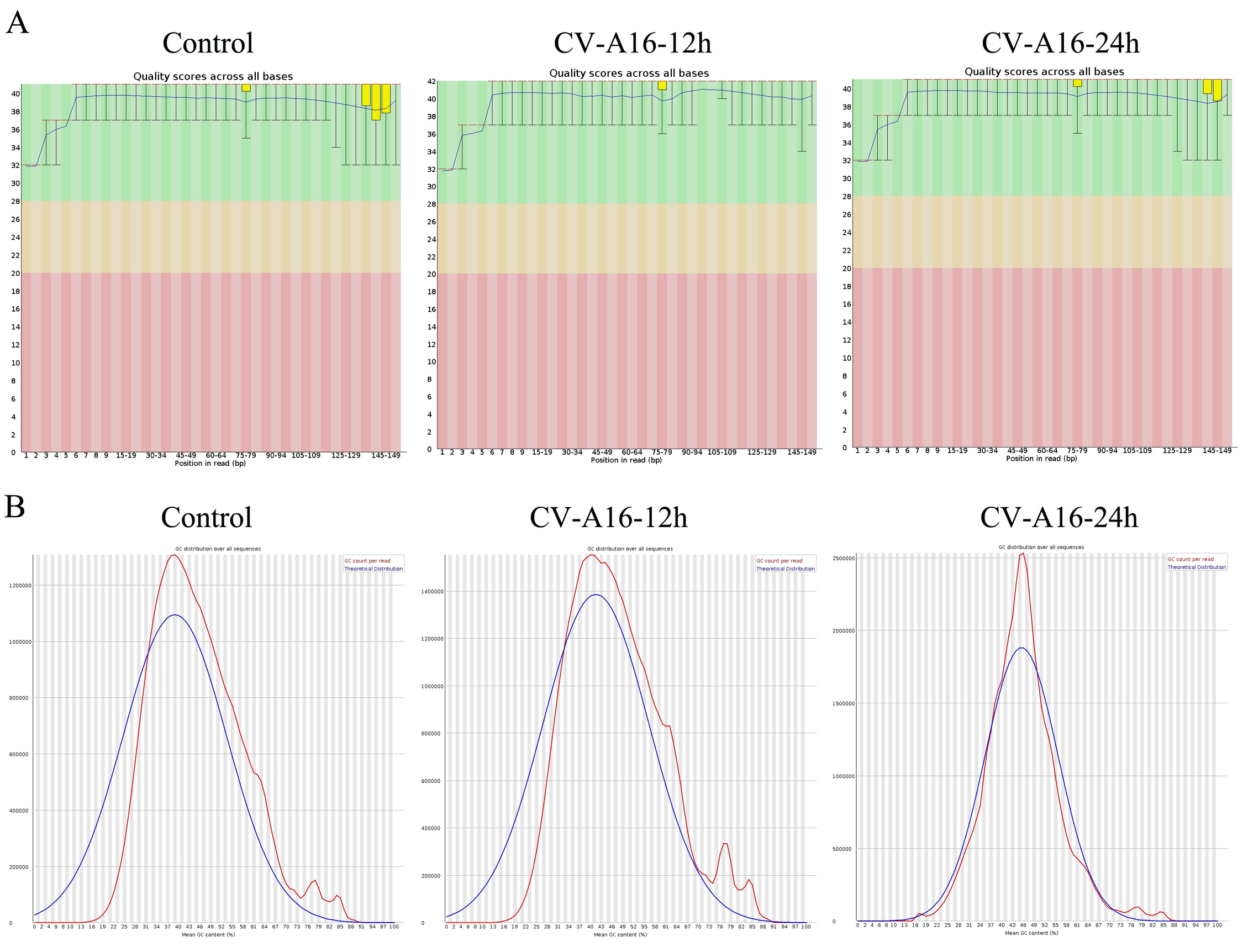

Supplement: S2 Fig — (A) QC results. (B) GC content. (TIF) [file pone.0241174.s002.tif]

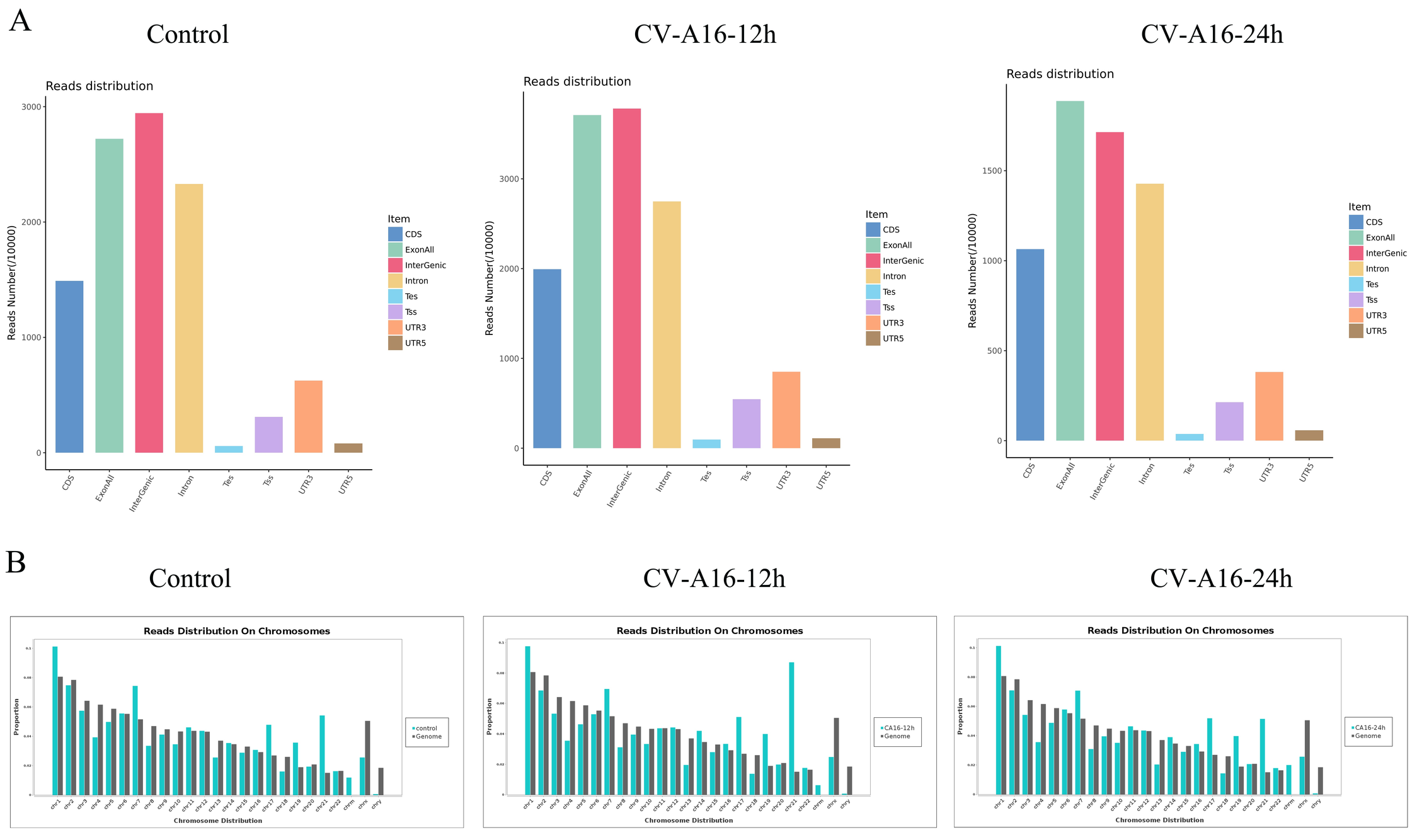

Supplement: S3 Fig — (A) Gene structure of dysregulated differentially expressed genes. (B) Chromosomal distribution of dysregulated differentially expressed genes. (TIF) [file pone.0241174.s003.tif]

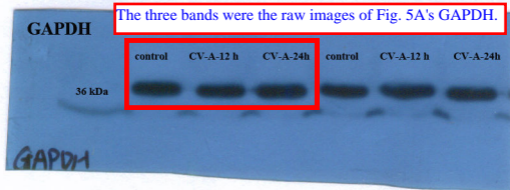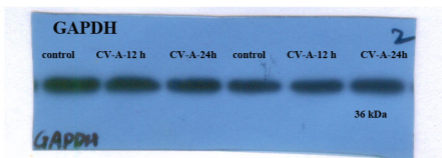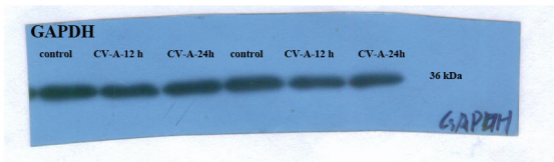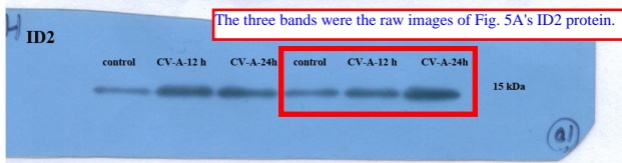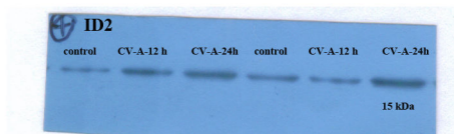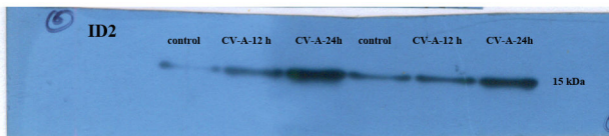

Supplement: S1 File — (PDF) [file pone.0241174.s006.pdf]
