# Supplementary material for: Comprehensive analysis of differential expression profiles via transcriptome sequencing in SH-SY5Y cells infected with CV-A16
Source: PLoS One. 2020 Nov 6;15(11):e0241174. doi: 10.1371/journal.pone.0241174 (PMC7647100; doi:10.1371/journal.pone.0241174)
Supplement: S1 Table — (DOCX) [file pone.0241174.s004.docx]

**S1 Table. Relevant information of gene and primer sequences for strand-specific qRT-PCR.**

| Gene Name | Primers |
| --- | --- |
| CDK6 | Forward primer: 5’-CTTCTCCAAGGAAACCGCCTA-3’  Reverse primer: 5’-CAAATGGCAGCACAATTGGTC-3’ |
| CHGA | Forward primer: 5’-CGTTGAGGTCATCTCCGACA-3’  Reverse primer: 5’- TCATCTTCAAAACCGCTGTGT-3’ |
| FOSB | Forward primer: 5’-AATGCTCCAGCTGTCGTCT-3’  Reverse primer: 5’-ACTCGCACCCAGAATTGTCA-3’ |
| COX2 | Forward primer: 5’-CCCACAGTCAAAGACACTCA-3’  Reverse primer: 5’-CTCATCACCCCACTCAGGA-3’ |
| TXNIP | Forward primer: 5’- CTTTTCGATAGTTTCGGGTCA-3’  Reverse primer: 5’- CCTAGTTTGTTAAATTGGCTCT-3’ |
| ZNF704 | Forward primer: 5’- TAGACAATTCCTGCCCGAAC-3’  Reverse primer: 5’- GTGCCAACTAATTCAGAACCC-3’ |
| GAPDH | Forward primer: 5’-TCTGACTTCAACAGCGACACC-3’  Reverse primer: 5’-TTGCTGTAGCCAAATTCGTT-3’ |
| CV-A16 (Used for the construction of standard RNA) | 5’-AACACTGAGGCTAGTAGTCAC-3’ (sense)  5’-CGTGTTTGATTCTCATGTACACC-3’(anti-sense) |
| CV-A16 (Used for qRT-PCR) | 5’-GTTTGTGAAAATGACGGACCC-3’ (sense)  5’-GTCATTTGCTTGAAGGTGCTC-3’(anti-sense)  Probe: FAM-CAGCTCAAGTGTCAGTCCCCT-TAMRA |
