# Supplementary material for: Comprehensive analysis of differential expression profiles via transcriptome sequencing in SH-SY5Y cells infected with CV-A16
Source: PLoS One. 2020 Nov 6;15(11):e0241174. doi: 10.1371/journal.pone.0241174 (PMC7647100; doi:10.1371/journal.pone.0241174)
Supplement: S2 Table — (DOCX) [file pone.0241174.s005.docx]

**S2 Table. The number of up-regulated and down-regulated genes in different gene types.**

| Groups | Gene type | The total number of differentially expressed genes | The number of up-regulated genes | The number of down-regulated genes |
| --- | --- | --- | --- | --- |
| CV-A16-12 h | tRNA | 12 | 1 | 11 |
|  | snRNA | 1 | - | 1 |
|  | snoRNA | 11 | 4 | 7 |
|  | RNase_P_RNA | 1 | 1 | - |
|  | RNase_MRP_RNA | 1 | 1 | - |
|  | pseudo | 8 | 2 | 6 |
|  | Protein-coding | 68 | 41 | 27 |
|  | Precursor_miRNA | 8 | 5 | 3 |
|  | ncRNA | 12 | 9 | 3 |
|  | miRNA | 14 | 8 | 6 |
| CV-A16-24 h | tRNA | 17 | 16 | 1 |
|  | snRNA | 9 | 5 | 4 |
|  | snoRNA | 22 | 2 | 20 |
|  | pseudo | 13 | 7 | 6 |
|  | Protein-coding | 61 | 31 | 30 |
|  | Precursor_miRNA | 11 | 3 | 8 |
|  | ncRNA | 6 | 5 | 1 |
|  | miscRNA | 2 | 1 | 1 |
|  | miRNA | 18 | 3 | 15 |
|  | Unknow | 2 | 1 | 1 |
