# Supplementary material for: Comprehensive analysis of differential expression profiles via transcriptome sequencing in SH-SY5Y cells infected with CV-A16
Source: PLoS One. 2020 Nov 6;15(11):e0241174. doi: 10.1371/journal.pone.0241174 (PMC7647100; doi:10.1371/journal.pone.0241174)
Supplement: Attachment — Submitted filename: Response to Reviewers.docx [file pone.0241174.s007.docx]

Dear Editors and Reviewers:

Thank you for your comments and suggestions concerning our manuscript entitled “*Comprehensive analysis of differential expression profiles via transcriptome sequencing in SH-SY5Y cells infected with CV-A16*” (Manuscript ID: PONE-D-20-10158). Those comments are all valuable and very helpful for revising and improving our paper, as well as the important guiding significance to our researches. We hope our revised manuscript meet with your approval.

In addition, we need to state here that we have followed the instructions of the magazine. Firstly, we logged into our account, located the manuscript record, and then checked for the action link “View Attachments”, but there were no attachment files to be viewed (See below). Therefore, we think there may be no reviewer’s comments, and there is no response to the reviewers’ comments in this reply.

Yours sincerely,

Yunhui Zhang, Corresponding author

Department of Respiratory Medicine, The First People’s Hospital of Yunnan province, Kunming, 650022, China.

E-mail: zhangyh123kh@163.com

**Response to editor’s comments**

Journal Requirements:

When submitting your revision, we need you to address these additional requirements.

1. Please ensure that your manuscript meets PLOS ONE's style requirements, including those for file naming. The PLOS ONE style templates can be found at https://journals.plos.org/plosone/s/file?id=wjVg/PLOSOne_formatting_sample_main_body.pdf and https://journals.plos.org/plosone/s/file?id=ba62/PLOSOne_formatting_sample_title_authors_affiliations.pdf

**Reply:** Thank you for your reminder. We will strictly follow the PLOS ONE’s style requirements.

2. We note that you are reporting an analysis of a microarray, next-generation sequencing, or deep sequencing data set. PLOS requires that authors comply with field-specific standards for preparation, recording, and deposition of data in repositories appropriate to their field. Please upload these data to a stable, public repository (such as ArrayExpress, Gene Expression Omnibus (GEO), DNA Data Bank of Japan (DDBJ), NCBI GenBank, NCBI Sequence Read Archive, or EMBL Nucleotide Sequence Database (ENA)). In your revised cover letter, please provide the relevant accession numbers that may be used to access these data. For a full list of recommended repositories, see http://journals.plos.org/plosone/s/data-availability#loc-omics or http://journals.plos.org/plosone/s/data-availability#loc-sequencing.

**Reply:** Sorry for this negligence. The sequencing data have been submitted to the Gene Expression Omnibus (GEO) database (www.ncbi.nlm.nih.gov/geo/) under the accession number GSM4593022 (Control group), GSM4593023 (CV-A16-12 h group) and GSM4593024 (CV-A16-24 h group).

Additional Editor Comments (if provided):

This paper by Hu and colleagues examines changes in the transcriptome of the human neuroblastoma cell line SH-SY5Y following infection with coxsackievirus A16 (CV-A16), one of the causative agents of hand-foot-and-mouth disease (HFMD) in children, but that has been also implicated with the development of severed neurological complications that can result in death.

The molecular bases whereby CV-A16 causes neurological symptoms are little understood, hence the significance of studies aimed at examining the impact of CV-A16 infection on the neuronal gene expression program. To this end the authors have used infection of SH-SY5Y cells as cell-based model.

The overall experimental design is straight forward and the authors have used appropriate RNAseq and bioinformatics tools to analyze changes in mRNA levels in CV-A16 infected SH-SY5Y, and use qRT-PCR on a group of selected genes to validate the results from RNAseq.

1. The main weakness of the paper is the lack of functional studies examining how the identified changes in host cellular gene expression in CV-A16 infected SH-SY5Y may contribute to neurological symptoms. Moreover, all the data presented relates to changes in RNA levels without showing whether these changes correlated with changes at the protein level, which would be the relevant ones in terms of mechanisms of neuropathology.

**Reply：**Thank you very much for your comments. In the transcriptome sequencing analysis of this article, it did not directly indicate which differentially expressed genes induced by CV-A16 infection may be the cause of neurological symptoms. But it can be concluded from the analysis of transcriptome sequencing that CV-A16 infection-induced changes in oxidation levels might be directly participated in the pathogenesis of CV-A16 infection. Moreover, among these differentially expressed genes in enriched “Gonadotropin-releasing hormone receptor pathway”, ID2 is not only associated with oxidative stress, but also with nervous system development. Early research showed that ID2 can promote the development of the nervous system^1^, and the latest research found that sustained high expression of ID2 can cause damage to the nervous system^2^. Therefore, the effect of ID2 on brain tissue is hailed as a “double-edged sword” and we also selected it to examine its protein levels. It was found that after CV-A16 infection, ID2 showed high expression in SH-SY5Y cells (Fig. 5A).

2. Another issue that needs additional discussion relates to the limitations of using a neuroblastoma cell line to examine the potential effects of CV-A16 on neuronal gene expression and associated neurological disturbances. It seems that the use of human iPSC derived neurons could provide a more relevant cell system, more so if glia cells are included in the experimental model of infection.

**Reply：**Thank you for your constructive advice. Actually, in many studies of neurotropic viruses, SH-SY5Y cells are often used as susceptible cells to explore their pathogenic mechanism *in vitro*. For instance, docosahexaenoic acid (DHA), an omega-3 polyunsaturated fatty acid, has a potential anti-inflammatory and neuroprotective effect against ZIKV infection in these neuron-like cells (these were SH-SY5Y cells)^3^; upregulation of antioxidants including SESN2 and, also, the xCT antiporter occurs to counteract the oxidative stress elicited by JEV infection in SH-SY5Y neuroblastoma cells^4^. Moreover, in a large number of previous studies, EV-A71, which is also the main pathogen of HFMD, also adopted SH-SY5Y cells to explore its potential neuropathic mechanism. For example, EV-A71 induced apoptosis of SH‑SY5Y cells through stimulation of endogenous let-7b expression^5^. Therefore, in this study, in order to explore the transcriptome changes after CV-A16 infected SH-SY5Y cells, we also selected SH-SY5Y cells as a susceptible nerve cell for research.

3. Figure S1 shows that CV-A16 induces a robust cytopathic effect at 24 hours post-infection and therefore many of the transcriptional changes observed may reflect changes associated with cellular death, and many of them might not be specific to CV-A16. It would be important to compare transcriptome changes caused by CV-A16 with those triggered by a non-viral cytotoxic stimulus.

**Reply：**Thank you for your suggestion. The effect of inducing cell death after viral infection is indeed not specific to the CV-A16 virus. Many viral infections will trigger cell death. In this paper, after analysis of transcriptome sequencing, the results showed that changes in oxidative stress may play an important role in the pathogenesis of CV-A16. Moreover, abnormal changes in ncRNAs may also be involved in the pathogenesis of CV-A16. Although CV-A16 infection triggered the formation of cell death, it was not clearly indicated in the transcriptome sequencing analysis. Therefore, in this article, we did not focus on the situation of virus infection-induced cell death.

**References:**

1	Andres-Barquin, P. J., Hernandez, M. C. & Israel, M. A. Id genes in nervous system development. *Histology and histopathology* **15**, 603-618, doi:10.14670/HH-15.603 (2000).

2	Jessen, K. R. & Mirsky, R. Negative regulation of myelination: relevance for development, injury, and demyelinating disease. *Glia* **56**, 1552-1565, doi:10.1002/glia.20761 (2008).

3	Braz-De-Melo, H. A. *et al.* Potential neuroprotective and anti-inflammatory effects provided by omega-3 (DHA) against Zika virus infection in human SH-SY5Y cells. *Sci Rep* **9**, 20119, doi:10.1038/s41598-019-56556-y (2019).

4	Carr, M. *et al.* Upregulated expression of the antioxidant sestrin 2 identified by transcriptomic analysis of Japanese encephalitis virus-infected SH-SY5Y neuroblastoma cells. *Virus Genes* **55**, 630-642, doi:10.1007/s11262-019-01683-x (2019).

5	Du, X. *et al.* Enterovirus 71 induces apoptosis of SHSY5Y human neuroblastoma cells through stimulation of endogenous microRNA let-7b expression. *Mol Med Rep* **12**, 953-959, doi:10.3892/mmr.2015.3482 (2015).
