# Supplementary material for: Comprehensive analysis of differential expression profiles via transcriptome sequencing in SH-SY5Y cells infected with CV-A16
Source: PLoS One. 2020 Nov 6;15(11):e0241174. doi: 10.1371/journal.pone.0241174 (PMC7647100; doi:10.1371/journal.pone.0241174)
Supplement: Attachment — Submitted filename: Response to Editor.docx [file pone.0241174.s008.docx]

Dear Editor:

Thank you for your guidance on our manuscript entitled “*Comprehensive analysis of differential expression profiles via transcriptome sequencing in SH-SY5Y cells infected with CV-A16*” (Manuscript ID: PONE-D-20-10158R2). We have resolved your issues according to your requirements. We hope our revised manuscript meet with your approval.

Most sincerely,

Yunhui Zhang, Corresponding author

Department of Respiratory Medicine, The First People’s Hospital of Yunnan province, Kunming, 650022, China.

E-mail: zhangyh123kh@163.com

**Response to Editor**

1. Please amend the title either on the online submission form or in your manuscript so that they are identical.

**Reply:** Thank you for your reminder. We have amended the title either on the online submission form or in your manuscript was identical.

2. Your ethics statement must appear in the Methods section of your manuscript. If your ethics statement is written in any section besides the Methods, please move it to the Methods section and delete it from any other section. Please also ensure that your ethics statement is included in your manuscript, as the ethics section of your online submission will not be published alongside your manuscript.

**Reply:** Thanks for your suggestion. We have supplemented our ethics statement in the Materials and methods section.

3. Please include a copy of Tables 1-3 which you refer to in your text on pages 45 and 48.

**Reply:** The Tables 1~4 have been inserted into the manuscript.

4. PLOS ONE now requires that authors provide the original uncropped and unadjusted images underlying all blot or gel results reported in a submission’s figures or Supporting Information files. This policy and the journal’s other requirements for blot/gel reporting and figure preparation are described in detail at https://journals.plos.org/plosone/s/figures#loc-blot-and-gel-reporting-requirements and https://journals.plos.org/plosone/s/figures#loc-preparing-figures-from-image-files. When you submit your revised manuscript, please ensure that your figures adhere fully to these guidelines and provide the original underlying images for all blot or gel data reported in your submission. See the following link for instructions on providing the original image data: https://journals.plos.org/plosone/s/figures#loc-original-images-for-blots-and-gels. In your cover letter, please note whether your blot/gel image data are in Supporting Information or posted at a public data repository, provide the repository URL if relevant, and provide specific details as to which raw blot/gel images, if any, are not available. Email us at plosone@plos.org if you have any questions.

**Reply:** According to the PLOS ONE requirements, the original images have been provided. And meanwhile, we have also stated in the cover letter.
