# Supplementary material for: Comprehensive analysis of differential expression profiles via transcriptome sequencing in SH-SY5Y cells infected with CV-A16
Source: PLoS One. 2020 Nov 6;15(11):e0241174. doi: 10.1371/journal.pone.0241174 (PMC7647100; doi:10.1371/journal.pone.0241174)
Supplement: Attachment — Submitted filename: Response to Editor.docx [file pone.0241174.s009.docx]

Dear Editor:

Thank you for your guidance on our manuscript entitled “*Comprehensive analysis of differential expression profiles via transcriptome sequencing in SH-SY5Y cells infected with CV-A16*” (Manuscript ID: PONE-D-20-10158R2). Regarding the question raised by the editor about the need to add some discussion about “the limitations of using a neuroblastoma cell line to infer viral induced changes in neuronal gene expression in the context of a whole organism”, we have actually given a corresponding response in the review comments of the second reply. And because we considered the latest review opinion reply as the third review opinion, so in the latest review opinion reply, there is no response to the question about “the limitations of using a neuroblastoma cell line to infer viral induced changes in neuronal gene expression in the context of a whole organism”. Here, we think we need additional explanation. Thus, we have responded to all the review comments one by one as follows. We hope our revised manuscript meet with your approval.

Most sincerely,

Yunhui Zhang, Corresponding author

Department of Respiratory Medicine, The First People’s Hospital of Yunnan province, Kunming, 650022, China.

E-mail: zhangyh123kh@163.com

**Response to Editor (Second review opinion)**

Additional Editor Comments (if provided):

The discussion section of the paper should incorporate a brief discussion about the limitations of using a neuroblastoma cell line to infer viral induced changes in neuronal gene expression in the context of a whole organism.

**Reply:** Thanks for your valuable advice. Actually, we all know that all *in vitro* cell model experiments cannot fully reflect a state *in vivo*. The *in vitro* cell model only provides a possible research direction for our research. This is a common limitation of all *in vitro* cell model experiments. However, in this study, we chose SH-SY5Y neuroblastoma cells first because SH-SY5Y cells are humanderived, and they express a number of human-specific proteins and protein isoforms that would not be inherently present in rodent primary cultures[1]; thereby some changes in SH-SY5Y cells may reflect certain conditions in the body. Additionally, SH-SY5Y cells are widely used in experimental neurological studies, including analysis of neuronal differentiation, metabolism, and function related to neurodegenerative and neuroadaptive processes, neurotoxicity, and neuroprotection, etc[2]. For example, neuron-like SH-SY5Y cells were applied to establish an in vitro cell model of Parkinson’s disease[3]. Moreover, SH-SY5Y cells are also often used in *in vitro* research models of neurotropic viruses, including Herpes simplex virus 1 (HSV-1) [4], Enterovirus D68 (EV-D68) [5], Rabies virus (RABV) [6], Japanese encephalitis virus (JEV) [7], West Nile virus (WNV) [8], and so on. Nevertheless, CV-A16 is also a neurotropic virus, and its mechanism of causing pathological changes in the nervous system has not been elucidated. Therefore, in this paper, we choose SH-SY5Y cells to construct an *in vitro* CV-A16 infection model to study the underlying neuropathic pathologic mechanism of CV-A16 infection. At the same time, it is undeniable that SH-SY5Y cells are only neuron-like cells, and they cannot completely represent the changes of neurons, nor can they represent the changes of the body when infected with CV-A16.

**Response to Editor (Third review opinion)**

1. Please amend the title either on the online submission form or in your manuscript so that they are identical.

**Reply:** Thank you for your reminder. We have amended the title either on the online submission form or in your manuscript was identical.

2. Your ethics statement must appear in the Methods section of your manuscript. If your ethics statement is written in any section besides the Methods, please move it to the Methods section and delete it from any other section. Please also ensure that your ethics statement is included in your manuscript, as the ethics section of your online submission will not be published alongside your manuscript.

**Reply:** Thanks for your suggestion. We have supplemented our ethics statement in the Materials and methods section.

3. Please include a copy of Tables 1-3 which you refer to in your text on pages 45 and 48.

**Reply:** The Tables 1~3 have been inserted into the manuscript.

4. PLOS ONE now requires that authors provide the original uncropped and unadjusted images underlying all blot or gel results reported in a submission’s figures or Supporting Information files. This policy and the journal’s other requirements for blot/gel reporting and figure preparation are described in detail at https://journals.plos.org/plosone/s/figures#loc-blot-and-gel-reporting-requirements and https://journals.plos.org/plosone/s/figures#loc-preparing-figures-from-image-files. When you submit your revised manuscript, please ensure that your figures adhere fully to these guidelines and provide the original underlying images for all blot or gel data reported in your submission. See the following link for instructions on providing the original image data: https://journals.plos.org/plosone/s/figures#loc-original-images-for-blots-and-gels. In your cover letter, please note whether your blot/gel image data are in Supporting Information or posted at a public data repository, provide the repository URL if relevant, and provide specific details as to which raw blot/gel images, if any, are not available. Email us at plosone@plos.org if you have any questions.

**Reply:** According to the PLOS ONE requirements, the original images have been provided. And meanwhile, we have also stated in the cover letter.

**References:**

1.	Kovalevich J, Langford D. Considerations for the use of SH-SY5Y neuroblastoma cells in neurobiology. Methods Mol Biol. 2013;1078:9-21. doi: 10.1007/978-1-62703-640-5_2. PubMed PMID: 23975817; PubMed Central PMCID: PMCPMC5127451.

2.	Xie HR, Hu LS, Li GY. SH-SY5Y human neuroblastoma cell line: in vitro cell model of dopaminergic neurons in Parkinson's disease. Chin Med J (Engl). 2010;123(8):1086-92. PubMed PMID: 20497720.

3.	Lopes FM, Schroder R, da Frota ML, Jr., Zanotto-Filho A, Muller CB, Pires AS, et al. Comparison between proliferative and neuron-like SH-SY5Y cells as an in vitro model for Parkinson disease studies. Brain Res. 2010;1337:85-94. doi: 10.1016/j.brainres.2010.03.102. PubMed PMID: 20380819.

4.	Shipley MM, Mangold CA, Kuny CV, Szpara ML. Differentiated Human SH-SY5Y Cells Provide a Reductionist Model of Herpes Simplex Virus 1 Neurotropism. J Virol. 2017;91(23). doi: 10.1128/JVI.00958-17. PubMed PMID: 28956768; PubMed Central PMCID: PMCPMC5686721.

5.	Brown DM, Hixon AM, Oldfield LM, Zhang Y, Novotny M, Wang W, et al. Contemporary Circulating Enterovirus D68 Strains Have Acquired the Capacity for Viral Entry and Replication in Human Neuronal Cells. mBio. 2018;9(5). doi: 10.1128/mBio.01954-18. PubMed PMID: 30327438; PubMed Central PMCID: PMCPMC6191546.

6.	Ahmad W, Li Y, Guo Y, Wang X, Duan M, Guan Z, et al. Rabies virus co-localizes with early (Rab5) and late (Rab7) endosomal proteins in neuronal and SH-SY5Y cells. Virol Sin. 2017;32(3):207-15. doi: 10.1007/s12250-017-3968-9. PubMed PMID: 28634871; PubMed Central PMCID: PMCPMC6598974.

7.	Song BH, Yun GN, Kim JK, Yun SI, Lee YM. Biological and genetic properties of SA(1)(4)-14-2, a live-attenuated Japanese encephalitis vaccine that is currently available for humans. J Microbiol. 2012;50(4):698-706. doi: 10.1007/s12275-012-2336-6. PubMed PMID: 22923123.

8.	Yang MR, Lee SR, Oh W, Lee EW, Yeh JY, Nah JJ, et al. West Nile virus capsid protein induces p53-mediated apoptosis via the sequestration of HDM2 to the nucleolus. Cell Microbiol. 2008;10(1):165-76. doi: 10.1111/j.1462-5822.2007.01027.x. PubMed PMID: 17697133; PubMed Central PMCID: PMCPMC7162166.
